# ‘Putting food on the table’: a critical discourse analysis of media representation of First Nations food insecurity in Australia

**DOI:** 10.1186/s12939-026-02764-8

**Published:** 2026-02-23

**Authors:** Leigh Bramwell, Leonie Cox, Danielle Gallegos

**Affiliations:** 1 School of Exercise and Nutrition Sciences, Queensland University of Technology (QUT), Victoria Park Road Kelvin Grove, Brisbane, QLD 4059 Australia; 2 Health Equity and Access Team, Metro South Health, McKechnie Drive Eight Mile Plains, Brisbane, QLD 4113 Australia; 3 School of Nursing, Queensland University of Technology (QUT), Victoria Park Road Kelvin Grove, Brisbane, QLD 4059 Australia; 4 Independent Researcher, Noosa Heads, Australia; 5 Centre for Childhood Nutrition Research, Faculty of Health, Queensland University of Technology (QUT), Graham St, South Brisbane, QLD 4101 Australia

## Abstract

Media discourses shape social values and public policy. Initial and ongoing colonisation in Australia results in disproportionately greater food insecurity experienced by First Nations peoples, negatively impacting health and wellbeing. This paper introduces that history and considers representations of food insecurity for First Nations communities in major Australian print and online news sources, reflecting on policy and practice implications. Examples of racialised media discourses are included in this paper and, while this content is subject to interrogation and critique, it may be distressing for First Nations readers. Using Critical Discourse Analysis, we interpreted five discourses: contradictory authentic Indigeneity, personal responsibility, moral failure, ‘save the children’, and food insecurity ‘solutions’. Our findings show the pervasiveness of the neoliberal values of individualism and personal responsibility, intersecting with racisms and perpetuating narratives of blame and dysfunction. Such perspectives distract from conversations about First Nations food security in terms of rights, food sovereignty, and the social determinants of health. Importantly, this media discourse analysis demonstrates a lack of focus on food insecurity experienced by First Nations peoples living in urban communities, with implications for action on the issue.

## Introduction

In the country now known as Australia, the food systems of First Nations peoples were and continue to be severely disrupted by colonisation, through colonisers forcibly displacing First Nations people from Country^1^, family and culture. Colonisers controlled Aboriginal peoples’ access to land and food, introducing salty, sweet and nutrient-poor rations that forced First Nations peoples into reliance on colonial systems, thus entrenching poverty [1]. When First Nations peoples here resisted colonial attempts to assimilate them, racist moralisation reinforced social views that they did not deserve charity, positioning them as the ‘undeserving poor’ [2]. Contemporary charitable responses to feeding people living in poverty are rooted in this history and reinforce similar attitudes. Being unable to feed yourself, family or community is imagined as an individual or racialised group failing, rather than arising from history and reflecting the current failure of government leadership and economic policy [3]. First Nations peoples continue to resist neocolonial decimation of food systems, through resourceful and contemporary connections to cultural foods [4, 5]. However, being unable to access affordable foods (food insecurity) is an escalating concern.

Food security exists “when all people, at all times, have physical, social and economic access to sufficient, safe and nutritious food that meets their dietary needs and food preferences for an active and healthy life” [6]. Food security for First Nations peoples living in remote^2^ areas of Australia is recognised, albeit with minimal action. However, food insecurity for First Nations peoples living in urban areas is rarely on the political agenda [7]. The latest national data – the first update in a decade – demonstrates an ongoing inequity with 41% of First Nations households impacted by food insecurity [8], compared to 13% across the broader Australian community [9]. The magnitude of food insecurity amongst First Nations communities is of national concern, yet data examining the experiences of urban First Nations peoples is limited [7]. Despite most First Nations peoples living in major cities and inner regional areas [10], food security research is usually conducted in remote or rural areas [11].

In advanced capitalist countries, food insecurity is a by-product of neoliberalism and economic inequality, rather than insufficient food production [12]. The lack of wages growth and failure of social protection payments to provide a living wage results in those on low incomes being more impacted by food insecurity [13]. The government’s refusal to address the structural barriers that perpetuate social inequality, relying on an insufficient and stigmatising charitable food system, arguably amounts to structural violence [3, 14].

Coherent cross-sectoral responses can improve societal responses to this complex issue [13], yet a range of perspectives informed by ill-founded assumptions about the causes of food insecurity abound [15, 16]. Instead of remaining focused on individual behaviours, action is needed to address the structural drivers of food insecurity, such as employment, housing and income [17]. There is a growing body of literature showing systemic racism is a determinant of food insecurity, with impacts regardless of socioeconomic factors [18, 19]. It compounds intersectional discrimination, including sexism, ableism, ageism and homophobia [18, 20].

This paper draws on a critical media analysis that formed part of a larger study of these matters. Here we consider how the news media frames food insecurity in relation to First Nations peoples and reflect on its impact on the social constructions of food insecurity, First Nations peoples, and consequent responses. This paper offers an exploration of the intersection between media discourses and First Nations food insecurity in Australia with a focus on representations of urban communities. We begin with an historical contextualisation of the matter during Australian colonisation and the use of food to control First Nations peoples. We then position our theoretical approach to media analysis, power and the role of media dominance in Australian society. We turn to an account of the study’s methodology, our position as researchers and the findings, finishing with a discussion of the social and political implications of these media discourses.

This paper includes racialised media and political discourse that may be distressing for First Nations peoples. Such content demonstrates racist views and how they perpetuate the ongoing trauma of colonisation. We interrogate this discourse, however its presentation here may be re-traumatising for some readers.

Our decisions about language intend to express respect and seek to challenge imbalanced relationships of power. ‘First Nations’ is used to respectfully acknowledge the hundreds of Aboriginal and Torres Strait Islander cultural groups that constitute the sovereign First Peoples of Australia. Aboriginal names are provided for Australian mainland locations where possible. Direct quotes are written verbatim from the original source. If they contain disrespectful language, such as ‘Indigenous’ written with a lowercase ‘i’, then [sic] acknowledges inappropriate content. Following the Diversity Council Australia [21] guidelines, ‘white’ is not capitalised to avoid reinforcing the power and privilege of white people.

In some sections, the literature mostly refers to Aboriginal people and communities, so at times the paper only refers to Aboriginal people. It is important to note that much of this literature is authored by non-Indigenous scholars. The historical context of colonial policies that morally maligned First Nations peoples in Australia has considerable relevance for this research, and further contextualises the current experience of food insecurity for First Nations peoples.

### Colonisation and food

Prior to European invasion, Aboriginal peoples in Australia had diverse diets and food practices that responded to local, seasonal availability [22]. Establishing relationships of “colonial benevolence and authority” [1], where food was used as a primary means of control, became central to relationships between Aboriginal people and colonisers. Following invasion and removal from Country, missions, cattle stations and police used rations of poor-quality staple food items to encourage assimilation, exploit cheap or free labour, and to protect colonisers [23]. For example, in Queensland following unsuccessful attempts to establish religious missions in the 1800s, the *Aboriginals Protection and Restriction of the Sale of Opium Act,* 1897 formalised the existence of missions and reserves. The Act legalised the removal and strict control of Aboriginal people into the middle of the twentieth century [24]. This ‘protection’ period nationally reflected the commitment to the moral reform of Aboriginal people, positioning them as “a child race” [24]. Consequently, it forced Aboriginal people into domestic service, institutions or missions, and subjected them to pervasive abuse and exploitation by colonisers [24]. Insufficient food across Aboriginal reserves and missions contributed to widespread ill-health [24]. O’Brien [2] describes hunger as experienced by Aboriginal people as “another form of physical violence”, employed to restrict movement and facilitate white possession of land.

Some colonisers viewed rations as a form of compensation for the dispossession of land from ‘the deserving poor’ [1]. However, European stereotypes of “the lazy native” fed fears that charity limited motivation to work, justifying practices where food became dependent on work [2]. In contrast, a discourse emerged at the time that acknowledged the inadequacy of rations as contributing to malnutrition, starvation, and general ill-health, and concerns about pauperism resulting from charity were widespread [2]. Racist views that Aboriginal people were “undeserving of better” underpinned a political culture of indifference and inevitability that acknowledged Aboriginal hunger while allowing it to continue [2]. In short, when not conforming to colonial ideas of moral worth, colonisers saw Aboriginal people as less deserving of charity [2].

### Media representation

Exploring the social construction of food insecurity and peoples’ status is central to understanding the development of perspectives and attitudes about the issue of food insecurity amongst First Nations peoples. We now consider the construction of First Nations food insecurity through Australian print and online news media.

The pervasive reach of the news media into every aspect of society is reflected in its relationship with governments, how it shapes dominant social values and what is considered knowledge [25]. Media ownership in Australia is among the most concentrated in the world, lower only than state-owned media interests in China and Egypt [26]. In terms of news media ownership, the Rupert Murdoch-owned News Corp accounted for just over half of the Australian readership in 2019–20 (51.9%), with independent media only contributing 7.4% [26]. Murdoch-owned media lobby government about key issues of corporate interest, affecting the careers of politicians and shaping public opinion through the power of messaging across their broad media platforms [27]. Manne [28] describes News Corp’s *The Australian* as “an unusually ideological paper, committed to advancing the causes of neoliberalism in economics and neoconservatism”. The context of Australian newspaper ownership, and the views presented by their authors, is central to interpreting this media analysis.

## Methodology

### Methodology and analytical framework

This media analysis is theoretically underpinned by social constructionism and critical discourse analysis (CDA). Both methodologies suggest that meaning is created through relationships and social structures, with language representing the social interactions that create it [29]. Socially constructed meaning is produced between people, within settings, and is specific to places and times [30].

CDA draws on its roots in critical theory, studying discourse and its relationship to power, social relations and inequities [31]. CDA examines social issues and the institutions and structures perpetuating imbalanced relations of power and power dominance through language [31]. This study employs Foucault’s [32] relational conceptualisation of power; that is, power is not an entity to be taken or gained but circulates as “a set of actions upon other actions” through knowledge and discourse. The news media exploits its dominant power position by socially constructing issues and social actors in particular ways [25]. Following Aldrich et al. [33] who argued that “solutions will depend on beliefs about cause”, the social representation of food insecurity by the media creates truths about First Nations communities with direct implications for policy decisions. Foucault [34] suggests that discourse is not only the mechanism by which power is exercised, but “it is the thing for which and by which there is struggle, discourse is the power which is to be seized”. CDA is useful in exploring media representations and their role in the social discourse that shape public perspectives. This study applies CDA as both the methodological framework and method for studying discourse and its social function [35].

### Researcher positionality

We identify as white, middle-class, cis-gendered women with professional backgrounds in humanities and health, and acknowledge our privilege as members of the dominant culture. We come to this research with personal views grounded in human rights, social justice and equity and apply a strengths-based approach. We collectively have extensive experience working with First Nations communities in health, education and research. We draw on the model of cultural safety that requires critical reflection on the part of professionals and services to oppose the impact of colonisation and its evolutions. Our concern is how the power, privilege, values, beliefs, attitudes and assumptions that researchers and health service cultures bring to encounters with people, influence practice, communication and outcomes [20, 36]. In this case, we are concerned with the relationship of these matters to media discourses.

### Context and setting

The larger study encompassing this critical media discourse analysis explores the social construction of food insecurity among staff working in urban First Nations primary health services. The CDA provided the broader sociocultural context of the specific health services in Meanjin/Brisbane, a highly urbanised capital city located on the north-eastern seaboard of Australia.

### Sampling method

Media database searches of Factiva and Australia & New Zealand Newsstream used various keywords depending on database functionality (Table 1). The search included state-based newspapers covering Meanjin/Brisbane, and national newspapers from major publishers. These databases included both online and print articles. Newspaper articles chosen as sources of text represent language carefully selected by publishers, having gone through an editorial process of determining word inclusion and exclusion. We included the National Indigenous Times website to access First Nations-specific media content. We considered other First Nations sources such as the Koori Mail, but had no search facility to identify relevant articles across many years.

**Table 1:** Database searches conducted for media analysis

Search terms	Database
(aborig* OR indigen*) AND (food NEAR/2 (access OR security OR insecurity OR availability OR poverty))	Australia & New Zealand Newsstream
australian and (aboriginal OR indigenous) and food security	Factiva
“food security”; “food access”	National Indigenous Times
(aborig* OR indigen*) AND poverty and Australia and food	Australia & New Zealand Newsstream

A date range from January 2007 to December 2016 applied to the databases searches. This range encompassed some key points in recent Australian history affecting First Nations peoples, including the 2007 Northern Territory Emergency Response^3^ (or ‘the Intervention’ as it is referred to here) and the 2008 Apology to Australia’s Indigenous Peoples. Pragmatically, we chosen a ten-year range to manage scope.

**Fig. 1:**
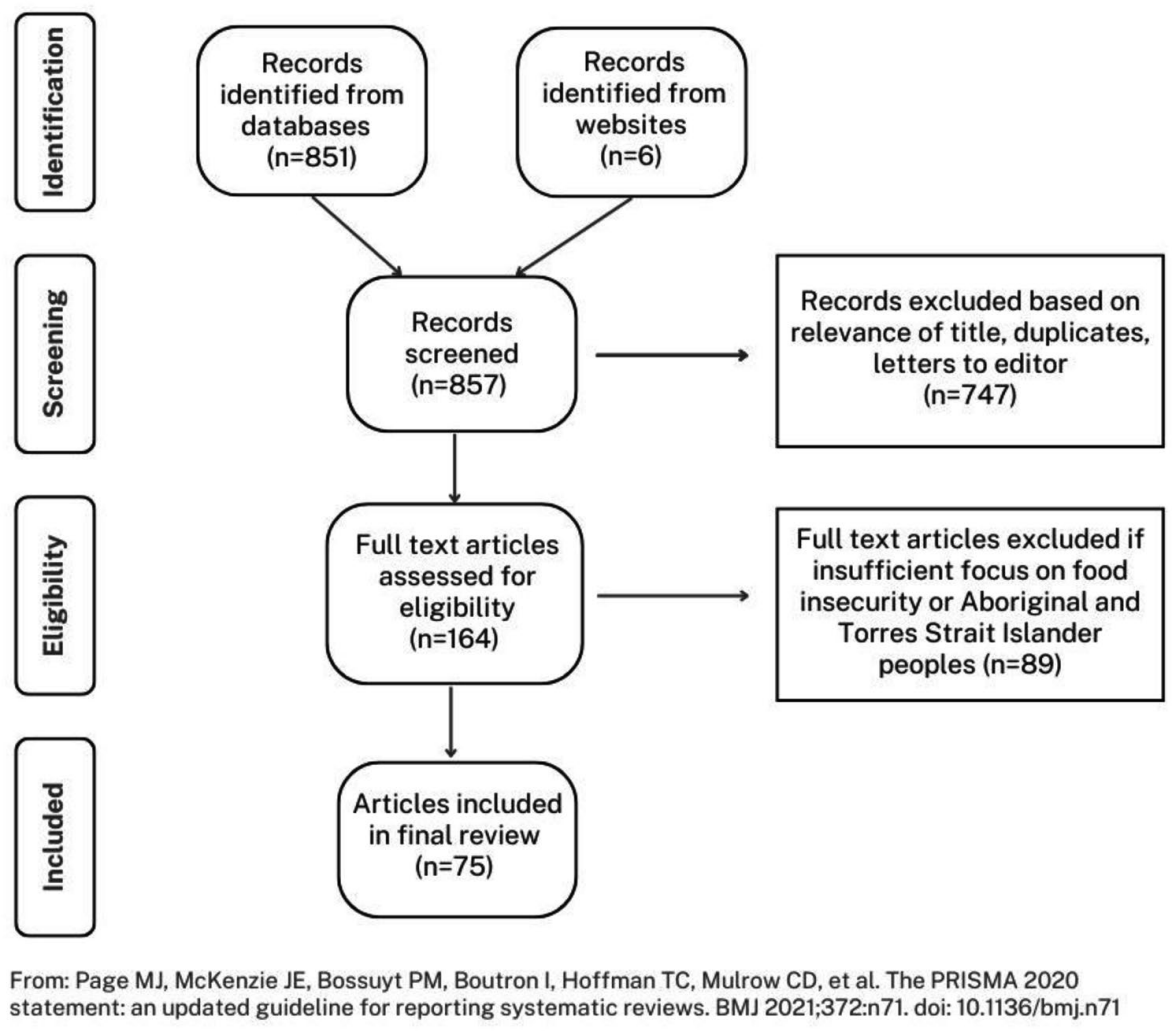
Sampling method – media discourse analysis

Initial screening of search results excluded articles based on the relevance of their title, then full text articles were assessed for suitability. This news media analysis aimed to identify discourses relating to First Nations food insecurity, so excluded articles with insufficient content relating to food insecurity (and related concepts, including food access or food supply) or to First Nations peoples. Articles selected had either a focus on food insecurity in the context of First Nations issues or a focus on First Nations issues where food was mentioned. For example, we excluded items with only a single reference to these concepts. The final sample contained 75 news articles, with the sampling method outlined in Fig. 1.

### Data analysis

**Fig. 2:**
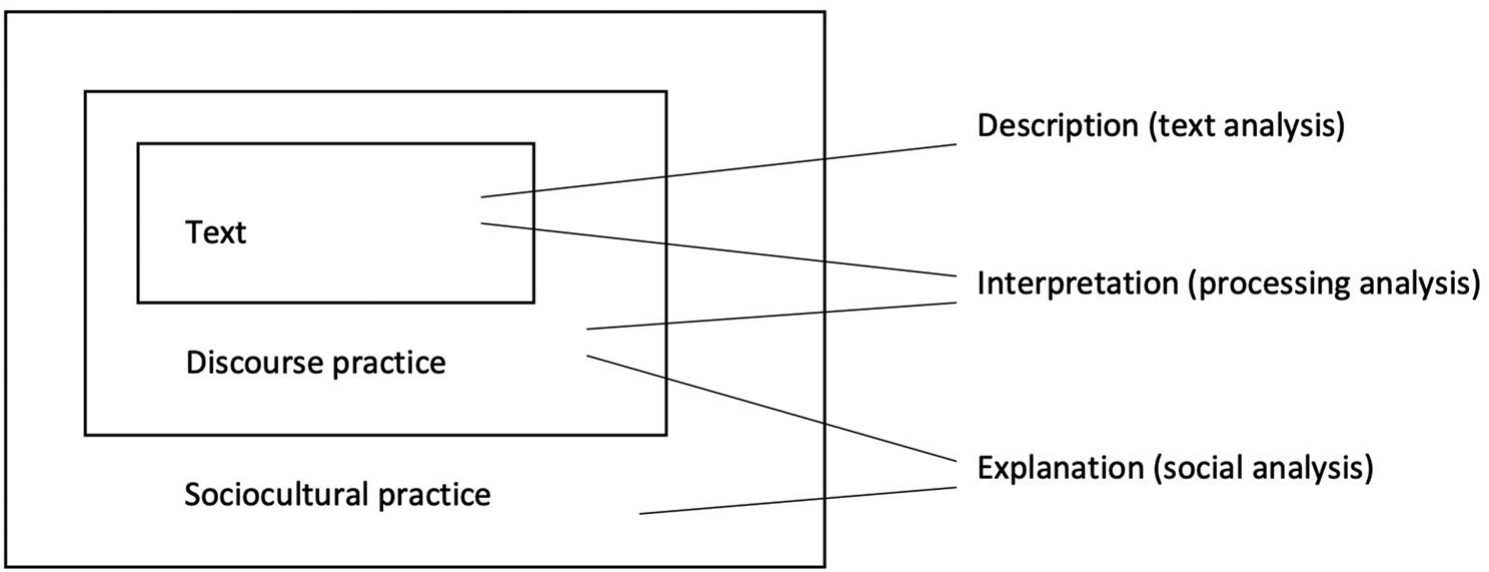
Fairclough’s three-dimensional method of critical discourse analysis. Note. source: Fairclough N. Critical discourse analysis: the critical study of language. 2nd ed. London: Longman; 2010

We used a multilayered CDA framework based on Fairclough’s work [31] (see Fig. 2), combining linguistic analysis with social theory.

Analysis included the meaning, and the form (structure and language) of the text itself with a critique of its social function and sociocultural context. This study drew on relevant questions from Fairclough’s [31] discourse analysis guide (see Table 2) along with their “desiderata for a critical analysis of media discourse” [25]. Initial analysis considered each layer of the CDA framework separately, with a particular focus on how power is employed within the texts. We then structured the analysis according to the discourses identified following a process of journaling, research team discussions and writing, with all three layers considered for each discourse.

**Table 2:** Questions guiding analysis

**Text analysis**
Semantic/grammatical relations between sentences and clauses: •What are the predominant semantic relations between sentences and clauses (causal – reason, consequence, purpose, conditional, temporal, additive, elaborative, contrastive/concessive)? •Are there higher-level semantic relations over larger stretches of text (e.g. problem-solution)? •Are particular relations of equivalence or difference set up in the text? Speech functions: •What are the predominant speech functions (statement, question, demand, offer)? •What types of statement are there (statements of fact, predictions, hypotheticals, evaluations)? Representation of social events: •What elements of represented social events are included or excluded, and which elements are more salient? •How abstractly or concretely are social events represented? •How are the social actors represented (activated/passivated, personal/impersonal, named/classified, specific/general)?
**Discourse analysis**
Genre: •Is the text characterised by a mix of genres? •What genres does the text draw upon and what are their characteristics? Difference: •What is the orientation to difference in the text (open recognition of difference, accentuation of difference/conflict, attempt to resolve difference, focus on commonality, consensus or normalisation of differences in power) Intertextuality: •Of relevant other texts/voices, which are included, which are significantly excluded? •How are other voices included? Attributed, quoted, indirectly reported? Assumptions: •What existential, propositional, or value assumptions are made? •Is there a case for seeing any assumptions as ideological? Discourses: •What discourses are drawn upon in the text, and how are they textured together? Is there a significant mixing of discourses? •What are the features that characterise the discourses that are drawn upon (semantic relations between words, collocations, metaphors, assumptions)?
**Sociocultural analysis**
Social events: •What social event, and what chain of social events, is the text a part of? •What social practice or network of social practices can the events be referred to, or seen as framed within? •Is the text part of a chain or network of texts?

*Note.* Source: Fairclough N. Critical Discourse Analysis: the critical study of language. 2nd ed. London: Longman; 2010

We acknowledge the potential tensions in adopting a method of analysis developed by a non-Indigenous scholar. McCartan et al. [37] suggest that CDA can be a useful tool in examining colonial power and the dominant structures that perpetuate inequities of First Nations peoples. However, white scholars must engage in critical self-reflection and “flip the narrative” by turning their attention to neocolonial power structures [37]. By drawing attention to the individualistic, negative and remote-focused media discourse in relation to First Nations food insecurity, we flip the narrative to include urban communities and the structural determinants. In doing so we draw on cultural safety, using critical self-reflection (where ‘self’ refers to persons, teams, services, systems and society) to interrogate white power and colonial structures. As Cox and Best [20] clarify, cultural safety entails ‘Ongoing cultural self-reflection by all … and systemic critical reflection by leaders in the organisations in which they work …’. This project was premised on the practice of this principal at all stages, and was realised through engagement with a community-led research governance committee and First Nations colleagues through conceptualisation to completion. Reflexivity and journalling by the lead author and constant team discussion supported these processes. The interpretation and conclusions reached challenge those involved in supporting food security amongst First Nations communities to also practice critical reflection to reconsider dominant discourses, entrenched stereotypes and victim blaming models.

## Findings

News Corp press accounted for two-thirds of the 75 media articles in this analysis, with only six articles from independent media including one Indigenous media organisation.

This media analysis interpreted five discourses: contradictory authentic Indigeneity, personal responsibility, moral failure, ‘save the children’, and food insecurity ‘solutions’. The discourses presented separately are not mutually exclusive, intersecting at many points discussed in this paper.

### Contradictory authentic Indigeneity discourse

All the articles in the media analysis focus on food insecurity amongst remote First Nations communities. This dominant discourse draws on representations of First Nations peoples living ‘traditional’ ways of life, juxtaposed against the ‘modern world’. Cohen’s [38] article states that it is unrealistic to think that Aboriginal people can continue “to hunt and fish as they have had for millennia, speaking their native tongue and communing with nature while at the same time earning a good living in a highly sophisticated, technically advanced society”. Some of the media articles position pre-colonial ‘traditional’ Aboriginal cultures as healthy, compared to representations of present-day communities experiencing “dysfunction” and “crisis” [39, 40].

The stark absence of urban representations of Indigeneity in the articles is consistent with the myth that “’real’ Aboriginal people don’t live in cities” [41]. First Nations peoples who do not conform to such stereotypical constructions consistently have their identities and legitimacy questioned [42]. Framing First Nations peoples as ‘savages’, living in remote communities in crisis, serves to justify the “colonial civilising mission” [43]. Such language is used in these media articles to portray ‘the Intervention’ as necessary, since “action was what was needed” [38] and discursively places Indigeneity only in remote locations [43]. Cohen’s representations of ‘traditional’ perpetuate the idea of primitive peoples “communing with nature” who cannot exist within a ‘superior’ capitalist, industrialised society.

Conventional constructions of First Nations peoples draw too on the notion of the ‘noble savage’ revering and erroneously assuming the existence of an untouched, non-urban Indigenous person uninfluenced by the ills of society [44]. The ‘noble savage’ frame relegates Indigeneity to the past, distancing First Nations peoples from equitable access to power and resources [44]. The colonial lens of modernity silences alternative knowledge systems that are not consistent with the dominant power structures [45].

Discourses invoking neoliberal concerns of the success and dominance of capitalism draw on the emotive notion of ‘taxpayers’ money’ to carry its critique of government support for remote Indigenous communities, and render them as incompatible with modern life. For example, Owen’s [46] article quantifies the amount of taxpayers’ money invested in one remote community to exemplify its lack of viability. They describe the community as “essentially a ghost town, where taxpayer-funded assets and infrastructure valued at about $20 million are going to waste”.

Reflecting similar neoliberal values, Neill and Owen’s [47] article quotes Aboriginal leader and representative for the Gold Coast Native Title Group, Wesley Aird:

where a community is either unable or unwilling to turn their lives around, then no amount of legislation can fix it, and the ethically responsible thing for government to do is to withdraw support. Falsely propping up a community is a socioeconomic form of palliative care.

Many articles in this analysis include Aboriginal voices to validate opinions about key issues. McQuire [48] argues *The Australian* newspaper uses “the Aboriginal leaders that most closely espouse its own viewpoints as a weapon to drown out any other form of debate, even in the face of evidence”. Aboriginal people whose values align with those of the newspaper are prominent and used to delegitimise alternative First Nations voices [48]. Here in the contradictory authentic Indigeneity discourse, Aird’s viewpoint is employed to strengthen the neoliberal position.

Descriptions of the impact of remoteness on food security in terms of food costs and infrastructure are frequent: “It’s hard enough to survive on welfare without food being 50% more expensive as it often is in remote communities, fresh food in particular” [49]. Shanahan [50] describes infrastructure issues in remote communities such as “only 6% of houses in Aboriginal communities have functioning fridges and adequate storage and bench space”. Chilcott’s [51] article states “Rampant health problems and poverty are being further entrenched in Aboriginal communities by unaffordable food prices” and “fresh food prices were crippling indigenous [sic] communities”. Although pointing to structural issues affecting remote communities, these comments contribute to representations of Aboriginal communities as unsustainable.

Instead of challenging the power relations and structures perpetuating poverty, a contradictory authentic Indigeneity discourse feeds the narrative that First Nations peoples are incapable of self-determination. It then justifies government intervention in the constructed crisis. The absence of representations of urban First Nations peoples has implications for drawing attention to and acting on urban issues, such as food insecurity.

### Personal responsibility discourse

Personal responsibility is a prevailing ideological goal across Australian politics, and most high-income countries. In his first speech then Prime Minister, Scott Morrison [52] stated “If you have a go in this country, you will get a go. There is a fair go for those who have a go”. Morrison’s overt articulation that ‘the fair go’ is conditional on effort implies deservedness for only some people in the community, and that failure is therefore an individual problem [53]. This personal responsibility discourse sits within a broader neoliberal narrative that applies to all Australians, however, in relation to First Nations peoples it is used to justify surveillance and intervention. The ‘personal responsibility’ media discourse is blatantly individualistic. It critiques how recipients spend their social security payments and justifies income management.

In the media analysis, social security is represented primarily as a pathway to employment “based on the principle the world owes no one a living and citizens must show responsibility in return for taxpayer support” [54]. The social welfare safety net is framed as “state-sponsored chronic welfare dependence” [55] when, from a neoliberal perspective, it should be “a hand up, not a handout” [56]. Statements such as “years of welfare dependency have demoralised communities to such an extent that few take responsibility for meeting their food needs” [57] express moral judgment and clearly link personal responsibility and food insecurity.

Welfare dependency is described as “sit-down money”^4^, “distributed with no strings attached”, resulting from a “permission culture that rewards dysfunction” and which has “poisoned the lives of successive generations of ill-educated people” [58]. While the author claims “the issues of concern have nothing to do with race, ethnic origin or cultural sensibilities”, they make clear comparisons with “dysfunctional remote Aboriginal communities” [58]. Disparaging representations of welfare dependency have a long history, with roots in early colonial notions of pauperism, the undeserving poor and reliance on charity. ‘Sit down money’ is now being used by conservative media as a derogatory term for ‘passive welfare’, blaming First Nations peoples for reliance on a colonial system that dispossessed and disempowered them.

Income management is framed in the articles as the necessary outcome for Aboriginal communities not taking responsibility for themselves [54, 56, 59–62]. Frequently claims that people spend money on drugs and alcohol flows onto praise for income management for having “put food on the table and taken grog off the streets” [61]. Force and control characterised some aspects of this discourse. For example, it quoted politicians as advocating to “fight passive welfare” [62]. Further, authors call for “pressure to be placed on irresponsible parents” [60], and system change that “demands parents take responsibility for their actions”, as “there must be limits to tolerance” [58]. The language of welfare dependency seeks to position the person receiving ‘welfare’ as a child [63], strengthening the narrative that they are ‘incapable’. Dependency is historically “racialised and gendered” with deeply negative moral connotations in neoliberal societies [63], enabling the justification of ‘necessary’ action to encourage personal responsibility.

Personal responsibility discourses draw heavily on negative ‘rights’ language, in comments like “what is needed is more responsibility. The rights-based agenda has gone too far” [58]. Similarly, ‘Food For Thought’ [59] justifies the cost of human rights by saying that “income management for remote Aborigines [sic] is not popular with the rights lobby, which places abstract ideals above the health of indigenous [sic] children – but it works”. The article suggests that “the challenge is less to increase the availability of nutritious foods than to encourage, compel if required, people to properly feed their children”, furthering the narrative that control is needed.

### Moral failure discourse

As mooted, a moral failure discourse reproduces stereotypical narratives of ‘dysfunctional’ First Nations peoples and communities, intersecting with poor parenting stereotypes. Emotive language is used to negatively frame First Nations peoples and communities, with words like “impoverished”, “vulnerable”, “poorest”, and “victims” [64]. This discourse is produced by describing communities as having “rundown and overcrowded houses”, “along dirt streets littered with rubbish and abandoned vehicles”, “containing blackened and stinking sewage” [56], with “shocking abuse of women and children” and “plagued by alcohol abuse” [54]. Aboriginal people and communities described as “starved” and “starving” [65, 66] constitutes their portrayal of being at “crisis level” and living “blighted” lives [65].

This media analysis demonstrates the ongoing creation of a moralising discourse about First Nations peoples spending social security money on drugs including alcohol. It is often cited by politicians as evidence of immoral and “irresponsible personal behaviour” that causes poverty [67]. Such irresponsible spending is presented as individual moral failure with claims that “compelling stores to sell fruit and vegetables is a waste of time if parents prefer to spend welfare payments on processed food, or worse, drink and drugs” [59]. The Ngaanyatjarra Pitjantjatjara Yankunytjatjara Women’s Council from Central Australia is quoted as saying a “significant proportion of community members, particularly men, have for decades been ‘blowing’ their benefits on non-essential items such as alcohol, illicit drugs and gambling” [47]. Here again, divergent Aboriginal voices are used to legitimise this discourse.^5^

There is considerable overlap between the ‘moral failure’ and ‘personal responsibility’ discourses, particularly in the way blame is shifted to First Nations peoples for the inequities experienced [33]. Deficit discourses that reinforce negative stereotypes of First Nations peoples are widely acknowledged by others [33, 68]. Fogarty [68] argues that “unhealthy, undereducated, unemployed, violent, and socially dysfunctional” tropes are discursively employed in narratives about First Nations peoples.

Moral framing is represented in this media analysis as justification for intervention and control, where a “serious government would intervene not just to limit access to alcohol or drugs, but to control diet and provide a smooth nutritional supply, minus sugar and minus fatty foods” [69]. Intersecting with the ‘save the children’ discourse, government rhetoric frames actions “to ensure that women and children had the right to live their lives free of violence” [54] as an “obligation to those children” [62]. ‘The Intervention’ is mostly positioned as being helpful in enabling First Nations households to pay for necessities. Senior federal politicians reinforce the narrative that income management has “helped families put more food on the table” [62] through the “quarantining of welfare payments so they could be spent on food rather than alcohol or gambling” [54].

The power of these negative representations is evident in their use to justify the sweeping state controls imposed solely on First Nations communities during ‘the Intervention’. Communities framed in terms of risk within a neoliberal context are exposed to more surveillance and control [70]. ‘the Intervention’ saw an escalation of entrenched paternalism and control by the Australian government over First Nations lives, resulting in suspension of the *Racial Discrimination Act 1975* in the name of addressing disadvantage and alleged abuse [70, 71].

### ‘Save the children’ discourse

The ‘save the children’ discourse evident in the media analysis centres on claims that First Nations children are in unsafe environments that include the risk of going without food. First Nations communities represented as places of child abuse reflects and perpetuates the spurious grounds justifying ‘the Intervention’. Reportedly, the Indigenous Affairs Minister in 2006 claimed child sexual abuse in remote communities when he stated “everybody in those communities knows who runs the paedophile rings” [56]. In the media articles, various government practices targeting First Nations communities framed as improving “safety for women and children” [60] claim that “vulnerable children are no doubt eating better and being better cared for as a result” [72]. Politicians’ voices feature heavily in this discourse drawing on narratives about the safety of children. Discourses connecting food and children’s welfare support misguiding government policies which “are determined to make sure children sleep safely at night, are healthy and well fed, and go to school each day” [73].

The ‘save the children’ discourse powerfully intersects with the moral failure discourse, describing “dysfunctional remote Aboriginal communities that caused uproar and an outpouring of concern for vulnerable children” [58]. Multiple discourses are reflected in this statement, reinforcing the social construction of dysfunction:

Of course, many communities are hard-scrabble places: anomie, drug use and near-universal welfare dependency remain the stubborn status quo. But surely if mothers could be persuaded to refrain from drinking, and domestic violence could be stamped out, and assured nutrition provided for parents and children – all feasible enough on paper as part of a concerted renovation scheme – then the syndrome’s grip might slacken: the factors that create the diabetes and renal disease pandemics would disappear.

[69]

The child protection system is discussed in relation to food in the media articles too. An article details a submission to the Northern Territory Child Protection Inquiry by child protection workers where they “called for aid to be delivered to starving Aboriginal children in remote communities” [74].

From colonisation to the present time, governments, their interconnected institutions, and the media employ representations of Aboriginal mothers as incapable of caring for their children, and households as places of neglect. These narratives justified assimilation through removal of thousands of First Nations children from their families; the Stolen Generations. Such portrayals and removal practices continue despite investigations since the 1970s into the human rights violations of removing Aboriginal children and placing them with white families [75]. In 2023, First Nations children were 10.8 times more likely to be in out-of-home care or on permanent care orders away from their family and less likely to be reunified with family than non-Indigenous children [76]. First Nations children being consistently overrepresented in child protection services creates new Stolen Generations.

The ‘save the children’ discourse and media commentary on the parenting of First Nations children prominently featured on a national breakfast television show. The segment followed an article in *The Daily Telegraph;* the opening line paraphrased the Federal Assistant Minister for Children and Families saying “White families should be allowed to adopt abused Aboriginal children to save them from rape, assault and neglect” [77]. The program then featured a panel of three white commentators discussing the article, where one person made the claim that “Just like the first Stolen Generation who were taken for their own wellbeing, we have to do it again perhaps” [78]. The ‘save the children’ discourse is built on a foundation of intervening in the lives of First Nations peoples ‘for their own good’. These shocking claims on national television not only show no awareness of current child removal practices; they also advocate for an intentional formal reinstatement of this deeply traumatic and devastating practice, entrenching devastating intergenerational impacts on families. A belief in racialised superiority underpins the notion that white people have the right to determine what is best for First Nations peoples.

### Food insecurity ‘solutions’ discourse

The ‘solutions’ discourse represents responses to food insecurity as being either the responsibility of governments or the individual, and maintains focus on remote communities. Accounts that “governments had an obligation” [51] reflect their responsibility where “People living in absolute poverty in remote areas require assistance from the state … to enable them to access what they need to survive” [79]. Other accounts represent freight and food subsidies as solutions to reduce healthy food costs and therefore improve health: “Aborigines [sic] need to be given tax cuts and subsidies for fresh food if the Government is to succeed in cutting the 17-year gap in life expectancy” [50].

Despite abundant reference to government responsibility within this discourse, there is often co-location of individualistic solutions to food security. Murphy’s [80] article began with the issue of fresh food affordability and the importance of subsidies to reduce freight costs, but then followed with the assertion that “Education programs were also needed to ensure outback children ate properly”. As discussed, income management features too as a solution to food insecurity through directly influencing individuals’ spending [59], [83]. Generally, the media sample echoes individualistic and moralistic responses to food insecurity, ignoring structural and historical drivers. Such discourses blame people for their social situation, removing responsibility from the state and society.

Simplistic, non-structural solutions to food insecurity appear in articles about community gardens in remote communities, within a context of government failure to listen and respond to community needs. A range of opinions exist, from community gardens being “an obvious community development strategy to combat this problem” [82], to conversely being seen as “a waste of money [that] will not reduce food prices” [83] and “rubbished by Aboriginal leaders” [81]. Responses espouse neoliberal values when the cost of initiatives to taxpayers is positioned as wasteful; “The gardens, which were part of an $800,000 food security program, included plantings of marjoram, thyme and coriander” [84].

For some commentators the solution is “as simple as providing refrigerators, offering healthy takeaway choices, subsidising freight on fresh fruit and vegetables and increasing the variety of healthy foods” [39]. While Simpson [39] does call for structural changes to address food security – “Australia must commit to system-level change processes that draw on Aboriginal communities as partners with a range of stakeholders” – the representation of solutions as ‘simple’ downplays the complexity of the structural change needed. In fact, the solutions must engage with a complex food system and the infrastructure that underpin food security. Central to these endeavours is addressing the unfinished business between First Nations peoples, the Australian state and the public.

## Concluding discussion

Culturally unsafe practice occurs when staff teams and services demean, diminish and disempower people [36], by unthinkingly drawing on media constructed stereotypes [71]. This process sustains their dominance in imbalanced relations of power and leads to poor outcomes for the people they are meant to serve. This critical discourse analysis of media representations of food insecurity related to First Nations peoples reflects the ongoing colonial project in Australia. Since food security and health are intrinsically linked, the constructions described in this paper highlight the historical connections that continue to enable the inequities experienced by First Nations peoples here.

A narrative of blame and dysfunction is continually reinforced. Dominant media and political discourses side-line the views of First Nations peoples [68], with negative representations indicative of a “subtle and underlying prejudice” [85]. This media analysis reflects McQuire’s [86] description of mainstream media as a “colonial apparatus … used to support the interests of the powerful at the expense of who they view as powerless”. The media discourses about First Nations communities clearly construct them as ‘places of neglect’. They claim abuse and dysfunction are rife, blaming people for their social situation, and linking food shortages to the deficits they construct. Notions of safety and the goal of putting food on the table justify ‘the Intervention’ and other methods of control over First Nations peoples. Multiple discourses positioning food insecurity as an individual problem call on people to ‘pull themselves out of poverty’. Neoliberal ideologies of mutual obligation and personal responsibility underpin much of the social commentary around food insecurity and social disadvantage amongst First Nations communities. Questions of deservedness are entwined with debates over ‘welfare’.

The role of structural factors and systemic barriers, including racism and colonisation, are mostly absent from the discourses identified in this analysis. The dominance of negative language towards recipients of social security payments, reflecting values such as ‘a hand up, not a handout’, show the pervasiveness of neoliberalism. This analysis argues that food is far from neutral, and having insufficient food is directly linked to historical and contemporary structural and social matters. The media’s negative social narrative exemplifies these matters, expressing institutionalised racism and carrying further stigma, social discrimination and risk for First Nations peoples. Our work aligns with Martens et al. [87] who argue too that food for First Nations communities is entwined with settler colonial relationships of power founded in control, food charity, and benevolence. The ongoing impact of colonisation on food insecurity is seen in Sherriff et al.’s [88] Australian study describing experiences of racism, poverty, and preferences for ‘mission foods’. To address food insecurity, Fredericks and Bradfield [89] suggest structural reform to invite critical reflection on white systems of power and the value of Indigenous peoples and knowledges.

In the media analysis, representations of food insecurity amongst urban First Nations communities are notably absent, with attention on remote communities and the logistical challenges of distance. There were no media articles focussing on urban First Nations communities in this sample, and where there was mention of urban communities it was not specific to First Nations communities. The absence of such representations constitutes a failure to construct First Nations’ urban food insecurity as a significant and unique issue. This failure has implications for the visibility and prioritisation of the matter for First Nations peoples, most of whom live in urban and regional communities.

The implications for urban-dwelling Aboriginal people are described by Behrendt [90]:

If rights are granted based on sympathy towards a particular stereotype, those Aboriginal people who do not fit within that paradigm will be excluded and considered inappropriate beneficiaries of these protections because they are not ‘authentic’; they will be seen as not ‘Real’ … And urban Aboriginal people, house-owning Aboriginal people, angry Aboriginal people, and Aboriginal people who do not speak their traditional languages will all fall outside of the Aboriginal people who are perceived to be deserving.

This thinking explains the media focus on food security in remote First Nations communities. Drawing on historical perspectives of the ‘deserving poor’, society implements responses to help those constructed as unable to help themselves. The absence of representations of urban First Nations peoples continues, rendered as the undeserving poor, hiding and minimising the food security issues they are facing.

A media analysis by van Burgel et al. [91] similarly found remote food insecurity in First Nations communities represented “as a simple issue requiring an immediate fix”, with underlying moral assumptions about the causes. Despite there being “no improvement in the cost of food in remote stores” [92], the numerous inquiries and policies relating to remote food security locate governments’ food insecurity focus. In contrast to the seemingly straightforward ‘solutions’ proposed for remote communities, urban food insecurity is constructed as an outcome of self-inflicted poverty engendering a very different response from media, politicians and services.

Ending poverty requires an ideological shift away from blaming individuals for their circumstances, towards acknowledging and addressing the complex systemic drivers of disadvantage and poverty. The media representations of remote and urban communities in this analysis imply that food security for First Nations peoples living in urban areas is subject to the inaccurate but predominant view held by the general Australian population. That is, feeding yourself and your family is a matter of personal responsibility. The narrative within these dominant discourses is that if you cook more, budget better, or get a job then you can provide food for yourself. And if those strategies fail, the band-aid solution of donated food from charities is acceptable. Continued advocacy to raise social security payments to adequate levels to address poverty and food insecurity [3] has failed to achieve that or overcome the dominance of this neoliberal narrative.

While this study explores the construct of food security, it sits within a broader discourse of food sovereignty. Indigenous food sovereignty grounded in decolonisation and self-determination moves beyond rights-based frameworks predicated on colonial systems [93]. For First Nations communities, food sovereignty enables control over food systems and restores connections to ancestral lands and waters impacted by colonisation and neoliberalism [93]. Dismantling the colonial power embedded in food systems, and acknowledging the interrelated sociocultural factors for First Nations peoples, is fundamental to achieving food sovereignty [87].

Despite the absence of representations in the mainstream media, urban First Nations communities are sources of power and pride, a matter overlooked in dominant representations of deficits and ill-health [94]. The identity of urban First Nations peoples is strong, arising from deep community social capital, but racism and urban cultural stereotypes challenge and question such strengths [95]. Drawing on the strengths of urban communities, and considering the unique context of urban areas, presents opportunities for self-determined responses to food insecurity in these areas.

## Limitations

CDA as a theoretical framework and method is critiqued for a lack of clarity around methods and processes [96]. There are claims that chosen tools are applied in ways that support a pre-existing ideological position [97]. We provided a detailed process of our analysis, selecting appropriate tools from the CDA ‘toolkit’ to suit the issue being studied, to examine relationships of power. Widdowson [98] questions the process of interpretation, including critical discourse analysts’ inability to disconnect themselves from the social world and their own socially constructed viewpoint. However, Fairclough [99] suggests that objective analysis – to describe “what is ‘there’ in the text” – is not possible without being influenced by the “’subjectivity’ of the analyst”. Qualitative analysis – indeed all interpretation – is done through the lens of the researcher. Our positionality described in this paper enables readers to gauge the influence of it on the analysis and interpretation.

## Data Availability

No datasets were generated or analysed during the current study.

## Footnotes

1 ‘Country’ refers to the lands, sky and waters that First Nations peoples are connected to through ancestral ties, and represents a complex sense of belonging and identity.

2 Remote, regional, and urban are terms used by the Australian Bureau of Statistics [100, 101] and within government to identify geographical areas in Australia. ‘Urban’ centres are determined by population density and infrastructure, and ‘regional’ and ‘remote’ areas are measured in geographical distance from these major centres. These terms do not describe how people live their lives. This work seeks to overcome the dominant discourse that positions ‘authentic’ Indigenous peoples as living in remote areas, and positions anyone not living in remote areas as inauthentic. This paper highlights and explores the lack of attention and visibility in non-remote areas that this dominant discourse produces.

3 The Howard Government’s 2007 Northern Territory Emergency Response, ‘the Intervention’ claimed it was in response to the *Ampe Akelvernemane Meke Mekarle “Little Children are Sacred”* report into child sexual abuse. Framed as a ‘national emergency’, it included legislative measures that severely restricted Aboriginal communities and suspended the *Racial Discrimination Act 1975* and *Native Title Act 1993*. Restrictions included compulsory government acquisition of Aboriginal-owned lands, mandatory quarantining of social security payments, restrictions on the sale and consumption of alcohol, and social security payments linked to school attendance. Human rights advocates considered it a return to assimilation and a violation of human rights imposed with no consultation. See Castan Centre for Human Rights Law Castan Centre For Human Rights Law [102] for a summary.

4 ‘Sit down money’ is now used to characterise the ‘personal responsibility’ discourse in relation to First Nations peoples, appropriating a term coined by Aboriginal communities in reference to unemployment benefits and popularised by the Warumpi Band’s eponymous song, a social commentary on the issues affecting Aboriginal communities. The keen observation in the term motivated the introduction of a government program that paid wages in return for work on community-determined projects [103].

5 While the gender dynamics arising from the women’s views are legitimate and worthy of further exploration, that is not our focus here. Rather what is of interest in this CDA work is how dominant discourses exploit these legitimate concerns.
